# Pasta, What a Feeling! A Multi‐Method Study on the Relationship Between Pasta Consumption and Happiness

**DOI:** 10.1002/fsn3.70240

**Published:** 2025-05-06

**Authors:** F. Grosso, A. Bonanomi, F. Pagnini

**Affiliations:** ^1^ Department of Psychology Università Cattolica del Sacro Cuore Milan Italy; ^2^ IRCCS Fondazione Don Carlo Gnocchi Milan Italy; ^3^ Department of Statistical Science Università Cattolica del Sacro Cuore Milan Italy

**Keywords:** ecological momentary assessment, emotions, happiness, implicit associations, Mediterranean diet, pasta consumption

## Abstract

The relationship between food consumption and emotions is complex and influenced by a variety of factors. Pasta, a staple of the Mediterranean diet, has not been extensively studied in terms of its emotional impact on consumers. This study aims to evaluate the emotions associated with pasta consumption, examining whether pasta consumption increases happiness levels and identifying individual characteristics that predict these reactions. A multi‐method approach was employed across two interconnected studies. Study 1 involved an online survey with a representative sample of 1532 Italians to assess explicit and implicit emotional associations with pasta. Study 2 monitored a subset of these participants (*n* = 83) over 2 weeks using ecological momentary assessment (EMA) to record emotions before and after meals, focusing on pasta consumption. Pasta consumption was consistently associated with positive emotions, particularly happiness, across different methods. Thematic analysis of open‐ended responses highlighted associations with family gatherings, warmth, comfort, and Italian traditions. Quantitative analyses revealed significant correlations between pasta appreciation and lower stress, higher quality of life, and greater mindfulness. Implicit Association Tests indicated a strong positive bias towards pasta‐related emotions. EMA data showed a significant positive effect of pasta consumption on happiness, especially when consumed in social settings. Pasta consumption is significantly linked to positive emotional experiences, particularly happiness, and is influenced by social and cultural contexts. These findings suggest that pasta can play a role in promoting psychological well‐being and highlight the importance of considering the emotional and social dimensions of food consumption in both health promotion and marketing strategies.

## Graphical abstract

Across two complementary studies, pasta consumption was found to be significantly associated with increased happiness, particularly when consumed in social settings. Implicit and explicit assessments showed strong positive emotional responses towards pasta, and mindfulness was found to enhance these effects. Cultural and contextual elements play a key role in shaping the emotional experience of food.

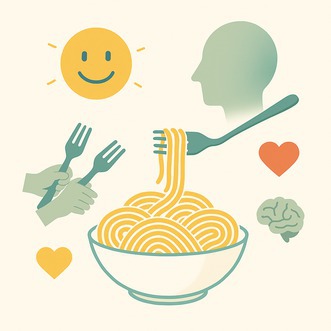

## 1 Introduction

The consumption of food generates various emotional reactions and is, in turn, influenced by the emotional experiences one undergoes. The relationship between food and emotions is quite complex and is influenced by factors such as hunger, satiety, age, expectations, and one's worldview (Köster and Mojet 2015). For instance, negative emotions like guilt and sadness influence individual preferences for carbohydrates, especially in the form of sugar (Lefebvre et al. 2019). On the other hand, specific dietary choices, whether aligned with one's worldview or not, can influence the emotions one experiences (Gibson et al. 2006). For example, consuming foods considered “healthy” leads to greater well‐being in those who endorse this choice (Wahl et al. 2017).

The Mediterranean diet, which has been recognized by UNESCO as an Intangible Cultural Heritage of Humanity (Trichopoulou 2021), has been extensively studied over the past few decades as a beneficial dietary pattern that combines health advantages with culinary enjoyment and conviviality (Donini et al. 2015). Adherence to a Mediterranean diet has not only been associated with physical health benefits (Guasch‐Ferré and Willett 2021), but also with psychological well‐being and generally positive emotions (Bracci et al. 2024). However, no data is available about how specific components of this diet would interact with psychological and affective states. Specifically, despite the existence of studies focusing on carbohydrate consumption and emotions (AlAmmar et al. 2020), there is limited information about the relationship between emotions and pasta. One study (Desmet and Schifferstein 2008) explored the emotions experienced by healthy individuals in response to tasting or consuming food, including pasta‐based meals. It was found that pleasant emotions (satisfaction, enjoyment, desire, and fun) were experienced more frequently than unpleasant ones (anger, sadness, and fear). A conference paper reported the results of a study about the psychophysiological responses following pasta consumption in a laboratory setting using neuromarketing techniques, reporting a positive cognitive state (Bellati et al. 2023). However, the small sample size and artificial research context limit the generalizability of the results.

The investigation into the role of emotions and emotional responses to food stimuli like pasta should consider the distinction between “experienced” emotions and “remembered” emotions (Kahneman and Riis 2005). Experienced emotions refer to the specific emotional experience at a given moment (i.e., “how do I feel right now”). Emotional associations, on the other hand, pertain to the perception of emotional tones expressed afterward (i.e., “What emotion do I associate with pasta”). The emotion felt during a particular situation may not necessarily correspond to the memory of that emotion. More general associations, such as those made regarding food, are more susceptible to cognitive biases and do not always reflect the emotional quality experienced “in reality” (Miron‐Shatz et al. 2009). Furthermore, an increasing interest has been developed over the role of “implicit” (i.e., non‐self‐reported and indirect affect measures) emotions elicited by food (Lagast et al. 2017). A comprehensive analysis about pasta consumption and emotional reactions should therefore integrate both perspectives.

## 2 Aims

This study aims to evaluate the emotions associated with pasta consumption, both in terms of direct experience and perception over time (i.e., remembered). In particular, the study will address the following research questions: (1) Which are the emotions associated with pasta consumption; (2) What is the emotional impact of pasta consumption (and, specifically, does pasta consumption increase *happiness* levels?); (3) What are the individual characteristics that predict *perceived* or *experienced* emotions related to pasta; and (4) What are the differences between pasta and other carbohydrate‐rich foods (e.g., rice) in terms of *emotional impact*. In data collected in the Italian context, we hypothesize that: (1) Pasta consumption is predominantly associated with positive emotions such as happiness (in this study, happiness refers to momentary and self‐reported emotional states); (2) Pasta consumption, particularly in social contexts, is associated with an increase in happiness; and (3) Higher mindfulness and lower stress are related to a stronger positive emotional impact of pasta.

## 3 Materials and Methods

To address these research questions, a multi‐method approach was employed with two partially interconnected studies. Study 1 consisted of a mixed‐method approach through an online survey with a representative sample of the national population from a panel to investigate explicit and implicit associations between emotions and pasta consumption. Study 2 involved a smaller number of participants, selected from those who participated in Study 1 as regular pasta consumers (at least three times a week, as self‐reported), and they were monitored using a dedicated SmartPhone application (RealLife Exp, Life Data Corporation, Marion, IN) for a total duration of 2 weeks. During these 2 weeks, participants provided specific information about the meals consumed and the emotions experienced during these meals. Both studies have been approved by the Ethics Committee of the Department of Psychology at UCSC (ref. 71–23). All participants provided informed consent prior to participation in the study.

### 3.1 Study 1

#### 3.1.1 Methods

Study 1 aimed to assess participants (*N* = 1532) in terms of their carbohydrate preference (i.e., pasta, rice, bread, cereals, cous‐cous) and create an emotional and personality profile, considering sociodemographic characteristics and emotions associated with carbohydrate consumption. Participants were recruited through a national marketing research agency using a stratified sampling method to ensure the representativeness of the Italian population in terms of age, gender, and geographic area. An online survey was conducted using the Qualtrics suite, employing various data collection tools, including open‐ended questions (qualitative approach) and rating scales to quantitatively measure respondents' opinions and behaviors. We collected data about the implicit associations between pasta and emotions. Specific questions and instruments were:

– Pasta consumption and preference for other carbohydrate sources (multiple choice): questions about how often pasta is consumed, how much it is enjoyed, their favorite pasta recipes, and other preferences in terms of carbohydrate foods.
– Emotions associated with pasta (open‐ended questions): images and emotions associated with pasta consumption and with different pasta recipes.
– Implicit association test (Task): based on the classic Implicit Association Test (Greenwald et al. 1998), this test aims to identify how strongly a person associates pasta with positive or negative emotions, even if they are not consciously aware of this association. Specifically, some keywords associated with pasta (e.g., “spaghetti,” “fusilli,” or “penne”) or other carbohydrates (e.g., “rice,” “bread,” or “pizza”) and others associated with emotions, either positive or negative (e.g., “happiness,” “guilt,” or “sadness”) were presented, and participants were asked to respond as quickly as possible to the test words by associating them with the reference categories. The task was inserted in Qualtrics with IATgen ShinyApp (Carpenter et al. 2019).
– Individual characteristics (questionnaires): mindfulness disposition, assessed with the Mindful Attention and Awareness Scale (Brown and Ryan 2003; Veneziani and Voci 2015); mindful eating propension, measured with the Mindful Eating Questionnaire (Clementi et al. 2017); positive and negative emotions experienced over the last month, assessed with the Positive and Negative Affective Schedule (Watson et al. 1988; Terraciano et al. 2003); personality traits, such as extraversion, agreeableness, conscientiousness, emotional instability, and openness to experience, with the short version of the Big Five Questionnaire (Rammstedt and John 2007; Guido et al. 2015); alexithymia, assessed with the Toronto Alexithymia Scale (Bagby et al. 1994; Bressi et al. 1996); the perceived stress level, assessed with the Perceived Stress Scale (Cohen et al. 1994; Mondo et al. 2021); and general quality of life, assessed with the single‐item McGill Quality of Life Scale (Cohen et al. 1999; Sguazzin et al. 2010); validated versions for the Italian population were used for all questionnaires.

#### 3.1.2 Analysis

Quantitative data were analyzed using descriptive statistics, including frequencies, means, and standard deviations, to summarize the key characteristics of the sample and study variables. Spearman's Rho was computed to examine the relationship between the considered outcomes, with significance levels set at *p* < 0.05. Statistical analyses were performed using SPSS (version 29). For the qualitative data obtained from open‐ended questions, a thematic analysis was conducted to identify and interpret patterns of meaning within the data. This analysis followed the six‐phase approach outlined by Braun and Clarke (2006). Two authors (F.G. and F.P.) independently coded the data and then collaborated to develop a consensus on the emerging themes. Discrepancies were resolved through discussion, ensuring a rigorous and reliable coding process. All statistical analyses in both studies were conducted by a statistician (A.B.) who was not involved in the study design and was blind to the study hypotheses.

#### 3.1.3 Results

One thousand five hundred thirty‐two people from the Italian population (*M* = 41.48; SD = 11.35) took part in an online survey (639 male, 892 female). The primary demographic data of the sample are presented in Table 1.

**TABLE 1:** Socio‐demographic characteristics and eating habits of the sample.

Gender [*n*, (%)]	
Male	639 (36.1)
Female	892 (50.4)
Non‐binary	4 (0.02)
Age [M, (SD)]	41.48 (11.35)
Educational level [*n*, (%)]	
Elementary school	2 (0.01)
Lower secondary school	131 (7.4)
High school	748 (42.2)
Bachelor's degree	254 (14.3)
Master's degree	300 (16.9)
Ph.D	101 (5.7)
Employment [*n*, (%)]	
Freelancer	164 (9.3)
Employee	891 (50.3)
Student	140 (7.9)
Unemployed	190 (10.7)
Retired	30 (1.7)
Income bracket [*n*, (%)]	
Less than €36,000	775 (43.8)
Up to €36,000	231 (13)
Between €36,151.99 and €70,000	310 (17.5)
Between €70,000 and €100,000	49 (2.8)
Prefer not to answer	171 (9.7)
Place of origin^a^	
Lombardy	282 (15.9)
Lazio	140 (7.9)
Campania	139 (7.8)
Marital status [*n*, (%)]	
Married	678 (38.3)
Cohabiting	270 (15.2)
Divorced	38 (2.1)
Separated	29 (1.6)
Single	514 (29)
Widowed	7 (0.04)
Physical activity level [*n*, (%)]	
No activity	352 (19.9)
Less than 30 min per day	659 (37.2)
At least 1 h per day	465 (26.3)
More than 2 h per day	60 (3.4)
Total	*N* = 1532
Special dietary regimen [*n*, (%)]	
Yes	119 (6.7)
No	1391 (78.5)
Only with a medical prescription	26 (1.5)
Total	*N* = 1536
Breakfast [*n*, (%)]	
Yes	1401 (79.1)
No	131 (7.4)

^a^ Only the three most represented regions are reported.

The sample shows a skewed preference towards pasta appreciation, and about half of the respondents (49.9%) consumed it daily. There was no specific preference for the type of pasta.

The spontaneous images that were most often associated with pasta were analyzed through thematic analysis, and the main topics were:

- Family (*N* = 560, 41%), including images of family gatherings, memories from happy reunions, and relatives, as well as family traditions.
- Positive emotions (*N* = 290, 21%), dealing with happy and warm images, a general sense of well‐being, and a positive outlook.
- Taste and flavor (*N* = 136, 10%), which highlight the sensory and pleasurable aspects of eating and cooking.
- Comfort and relaxation (*N* = 91, 7%), which describe images of calmness, security, leisure, and relaxation.
- Tradition and culture (*N* = 75, 6%), encompassing content like Italian culture, traditional recipes, and the Mediterranean diet.
- Food and nutrition (*N* = 70, 5%), referring to food associations (e.g., condiments), eating patterns, and dietary preferences.
- Health and well‐being (*N* = 22, 2%), which include health and nutritional benefits, sports, and a positive impact on both mind and body.
- Other (*N* = 109, 8%), when the responses did not fit neatly into the above categories.

As for the question “Imagine you have just eaten a plate of pasta. What emotions and sensations do you imagine you are feeling?”, the answers fit into these main themes:

- Satiety (*N* = 462, 34%), which includes responses that describe a feeling of fullness or being satisfied with the quantity of food consumed.
- Satisfaction (*N* = 394, 29%), with responses expressing pleasure and contentment with the meal, often highlights the enjoyable taste and experience.
- Happiness (*N* = 137, 10%), reflecting feelings of happiness, joy, and positive emotional states resulting from the meal.
- Taste (*N* = 30, 2%), which includes responses that specifically mention the taste or flavor of the food.
- Other (*N* = 330, 24%), referring to responses that do not align clearly with the main identified themes.

The analysis of the implicit associations (*N* = 501) indicates the presence of a positive cognitive bias (*d* = 1.49, *t* = 33.469, *p* < 0.001) toward the associations between pasta and positive emotions.

Overall, pasta appreciation was associated (Table 2) with fewer negative emotions, positive implicit associations, a better level of well‐being, lower stress, and increased mindfulness (both in terms of general mindfulness and mindful eating).

**TABLE 2:** Correlations between how much people like pasta and psychological characteristics.

	How much do you like pasta	
Positive emotions		
rho‐S	0.036	
*p*‐value	0.195	
Negative emotions		***
rho‐S	−0.109	
*p*‐value	< 0.001	
Positive implicit associations		**
rho‐S	0.146	
*p*‐value	0.001	
Quality of life		***
rho‐S	0.139	
*p*‐value	< 0.001	
Stress		*
rho‐S	−0.059	
*p*‐value	0.030	
Mindful eating		***
rho‐S	0.115	
*p*‐value	< 0.001	
Alexithymia		*
rho‐S	−0.064	
*p*‐value	0.020	
Mindfulness		***
rho‐S	0.115	
*p*‐value	< 0.001	

* *p* < 0.05.

** *p* < 0.01.

*** *p* < 0.001.

### 3.2 Study 2

#### 3.2.1 Methods

Participants in Study 2 were selected from the initial sample based on their self‐reported consumption of pasta at least three times per week and their willingness to take part in a two‐week experience sampling methodology (EMA). This threshold was set to ensure a sufficient number of pasta‐related meal entries. They were monitored for 2 weeks with an EMA (Van Berkel et al. 2017), receiving a phone notification before and after each meal, rating selected emotions and feelings (on a scale from 1 to 10): hunger, happiness, sadness, tiredness, and anger. In the post‐meal assessments, they were also asked to report what food they had, how much they liked it, and what the setting was (either alone, with someone, at home or at work). To address common EMA challenges like missing data and low compliance, participants received automated reminders and monetary incentives. Only complete pre‐ and post‐meal entries were analyzed.

#### 3.2.2 Analysis

We employed a Gaussian Linear Mixed Model (GLMM) to analyze the effect of pasta consumption on changes in happiness levels (Δ_Happiness). GLMM handles clustered data, such as repeated measures on subjects over time or experimental units grouped by treatment, recognizing the dependency within clusters. Moreover, GLMM integrates fixed effects with random effects. In our study, the dependent variable, Δ_Happiness, represents the change in self‐reported happiness before and after lunch or dinner. Happiness was measured on a 10‐point scale ranging from 1 to 10. The dependent variable Δ_Happiness was measured on a 19‐point scale ranging from −9 to 9, where −9 represents the maximum negative happiness differential, while 9 represents the maximum positive happiness differential. 0 indicates no difference in happiness before and after the meal. The fixed effect was pasta consumption, a dichotomous variable (0 = no pasta, 1 = pasta). Participants were included as a random effect to account for individual differences. The model can be expressed as:

$$\Delta \_{\text{Happiness}}_{\mathrm{ij}}=\beta_{0}+\beta_{1}{\text{Pasta}}_{\mathrm{ij}}+u_{0i}+\varepsilon_{\mathrm{ij}}$$

where $\beta_{0}$ is the intercept, $\beta_{1}$ is the fixed effect of pasta consumption, $u_{0i}$ is the random intercept for participant *i*, and $\varepsilon_{\mathrm{ij}}$ is the normally distributed residual error.

The model was fitted using the GAMLj package in R, implemented in Jamovi 2.5.3. The restricted maximum likelihood (REML) method was used for parameter estimation. We conducted additional sensitivity analyses to test the robustness of our findings. Specifically, we estimated an alternative mixed‐effects model that included income, education level, and overall dietary habits as covariates. As these variables did not significantly improve model fit or alter the significance of the main effect, and in the interest of model parsimony, they were not included in the final or subsequent models.

#### 3.2.3 Results

Eighty‐three participants joined the study, providing information about 1843 overall valid sessions, divided into 920 lunch and 923 overall dinner sessions.

The fixed effect of pasta (Table 3) consumption on Δ_Happiness was significant ($\beta_{1}$ = 0.316 [95% CI: 0.168–0.464], SE = 0.076, *p* < 0.001). The random intercept variance was estimated at 0.137, indicating considerable variability in baseline happiness levels across participants. Overall, the fixed effects omnibus test was significant (*F* = 17.507, df = 1, *p* < 0.001). The model fit was assessed using the Akaike Information Criterion (AIC = 6220.768), the Bayesian Information Criterion (BIC = 6242.768), and the log‐likelihood (LL = −3106.384).

**TABLE 3:** Parameter estimates (Fixed coefficients)—GLMM with a fixed effect (Pasta) and a random effect (Participants).

Names	Effect	Estimate	SE	95% confidence intervals		df	*t*	*p*
				Lower	Upper			
Intercept	Intercept	0.152	0.068	0.019	0.285	49.482	2.239	0.030
Pasta	1–0	0.316	0.076	0.168	0.464	1805.570	4.184	< 0.001

**FIGURE 1:**
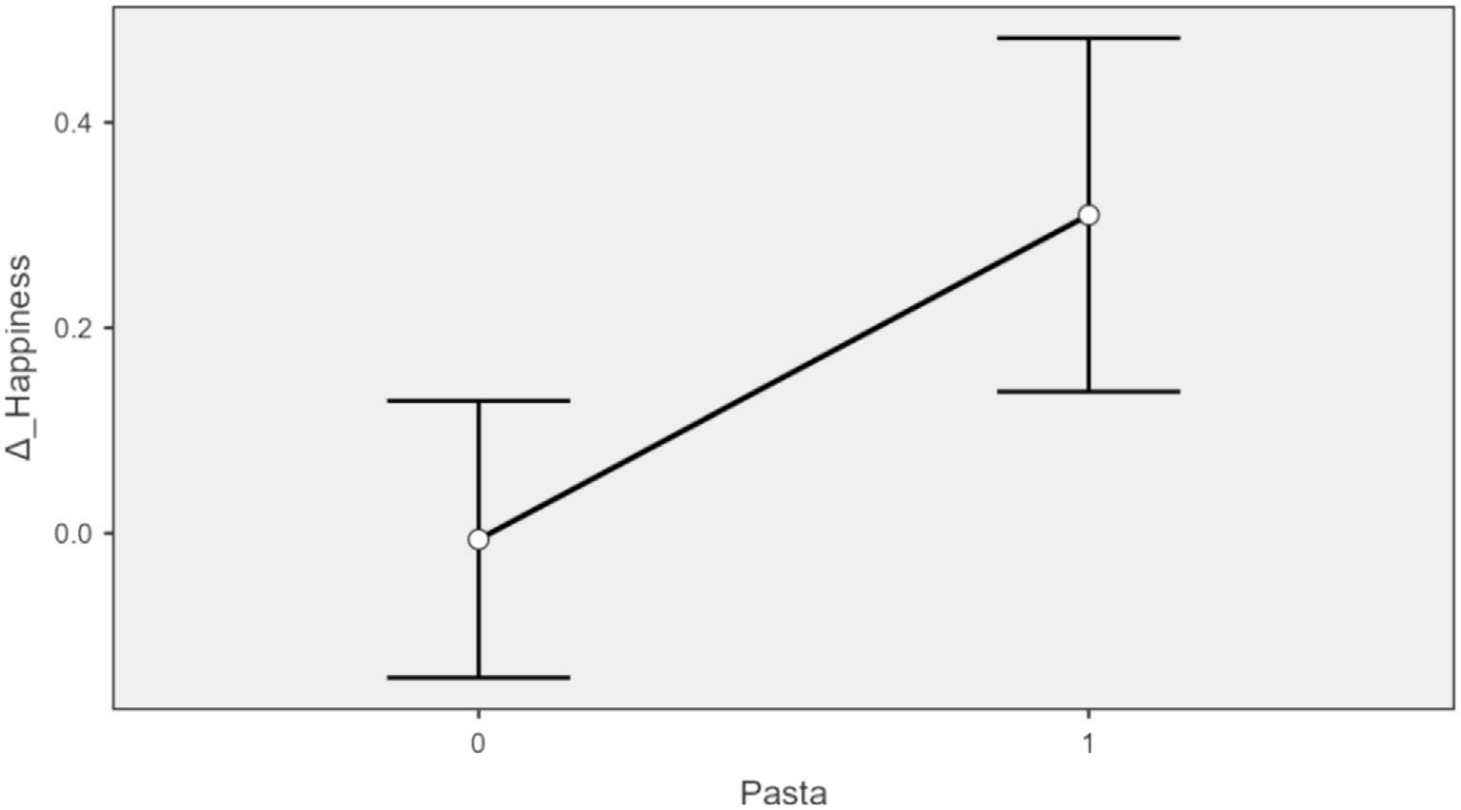
Estimated marginal effects (0 = no pasta consumption; 1 = pasta consumption).

Figure 1 reports the plot of the estimated marginal effects of the fixed effect pasta consumption (Random effects were plotted across participants). When the same model was applied to other meal choices, no significant results were observed.

The significant positive effect of pasta consumption on variation of Happiness suggests that participants who consume pasta reported greater increases in happiness compared to those who did not. The random intercept variance underscores the importance of accounting for individual differences in baseline happiness levels. The model fit indices indicate that the model provides a reasonable fit to the data.

We tested other GLMMs to verify the presence of a relationship between happiness variation and the interaction between pasta consumption and a series of variables of different nature. Firstly, with socio‐demographic variables (gender, age, marital status), then with behavioral variables (time of day when pasta was consumed, environmental situation, company, enjoyment of the pasta), and psychological variables (collected during Study 1) such as personality traits, positive and negative affect, mindful eating, perceived stress, and mindfulness disposition.

Regarding the socio‐demographic variables, no relationship emerged either for the main fixed effect considered each time or for an interaction effect with pasta consumption. The same result emerged with the psychological variables considered, except for a significant main effect of the mindfulness scale. There is also a slight trend for the interaction effect, but it is not significant. Table 4 reports the estimates of the GLMM constructed with two main fixed effects (pasta consumption and mindfulness score), an interaction effect (between pasta and mindfulness), and participants were included as a random effect to account for individual differences.

**TABLE 4:** Parameter estimates (Fixed coefficients)—GLMM with two fixed effects (Pasta and MAAS and their interaction) and a random effect (Participants).

Names	Effect	Estimate	SE	95% CI		df	*t*	*p*
				Lower	Upper			
(Intercept)	(Intercept)	0.291	0.113	0.069	0.512	130.596	2.577	0.011
Pasta	1–0	0.499	0.191	0.124	0.874	374.245	2.610	0.009
MAAS	MAAS	0.506	0.231	0.052	0.960	36.145	2.188	0.035
Pasta × MAAS	(1–0) × MAAS	0.466	0.326	−0.175	1.106	638.842	1.427	0.154

The significant positive effect of pasta consumption (*p* = 0.009) and mindfulness (*p* = 0.035) on variation of Happiness suggests that participants who consumed pasta reported greater increases in happiness compared to those who did not, as well as those with higher mindfulness scores. The interaction effect is not significant (*p* = 0.154) but shows a slight trend: the happiness variation between those who consume pasta compared to those who do not increases as mindfulness values increase. A strong interaction effect between pasta consumption and where pasta is consumed emerges instead. Table 5 reports the estimates of the GLMM constructed with two main fixed effects (pasta consumption and place), an interaction effect (between pasta and the place), and participants were included as a random effect to account for individual differences. The fixed effect “Where are you” is a categorial variable on 4 modalities, at home (reference category), at work, together and alone.

**TABLE 5:** Parameter estimates (Fixed coefficients)—GLMM with two fixed effects (Pasta and Place and their interaction) and a random effect (Participants).

Names	Effect	Estimate	SE	95% CI		df	*t*	*p*
				Lower	Upper			
(Intercept)	(Intercept)	0.076	0.123	−0.166	0.318	283.303	0.616	0.538
Pasta	1–0	−0.103	0.214	−0.522	0.317	1284.071	−0.480	0.631
Place 1	At work—at home	−0.239	0.367	−0.958	0.480	1461.321	−0.652	0.514
Place 2	Togheter—at home	0.067	0.129	−0.185	0.320	1592.356	0.524	0.601
Place 3	Alone—at home	−0.146	0.213	−0.564	0.272	995.908	−0.684	0.494
Pasta × Place 1	(1–0) × (at work—at home)	−1.390	0.713	−2.788	0.009	1584.064	−1.949	0.051
Pasta 1 × Place 2	(1–0) × (togheter—at home)	0.562	0.251	0.069	1.056	1694.798	2.237	0.025
Pasta 1 × Place 3	(1–0) × (alone—at home)	−0.735	0.413	−1.545	0.076	1172.649	−1.777	0.076

The model can be expressed as:

$$\begin{array}{c} \Delta \_{\text{Happiness}}_{\mathrm{ij}}=\beta_{0}+\beta_{1}{\text{Pasta}}_{\mathrm{ij}}\\ +\beta_{2}{\text{Place}}_{\mathrm{ij}}+\beta_{3}{\text{Pasta}}_{\mathrm{ij}}\times {\text{Place}}_{\mathrm{ij}}+u_{0i}+\varepsilon_{\mathrm{ij}} \end{array}$$

where $\beta_{0}$ is the intercept, $\beta_{1}$ is the fixed effect of pasta consumption, $\beta_{2}$ is the fixed effect of where pasta is consumed, $\beta_{3}$ is the fixed effect of the interaction between pasta consumption and place, $u_{0i}$ is the random intercept for participant *i*, and $\varepsilon_{\mathrm{ij}}$ is the residual error.

With the introduction of the fixed effect related to the interaction between pasta consumption and where it is consumed, the main fixed effect of the pasta consumption is not significant (*p* = 0.631) while the interaction effect is highly significant, particularly when pasta consumption occurs together compared to when it happens at home ($\beta_{3}$ = 0.562, *p* = 0.025). The random intercept variance was estimated at 0.147, indicating considerable variability in baseline happiness levels across participants. Overall, the fixed effects omnibus test was significant only for the interaction effect (*F* = 4.607, df = 3, *p* = 0.003). A sensitivity analysis including income, education, and dietary habits as covariates was conducted. The main effect of pasta consumption on well‐being remained statistically significant, although slightly attenuated. The additional covariates were not significant predictors, and model fit indices showed only marginal improvement. For these reasons, and to preserve clarity and parsimony, the final and subsequent models excluded these covariates.

**FIGURE 2:**
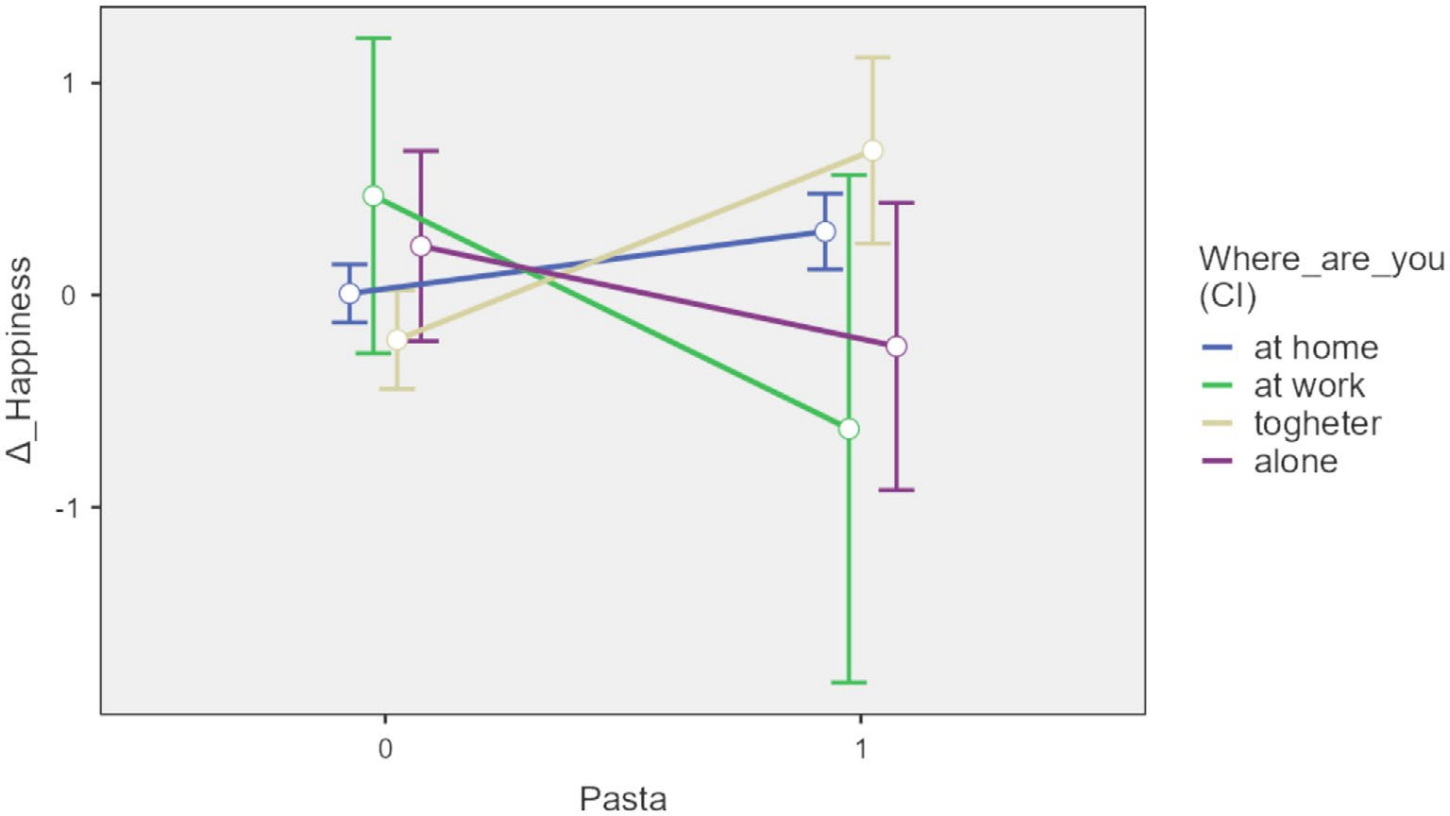
Estimated marginal effects, GLMM with interaction between pasta and place (0 = no pasta consumption; 1 = pasta consumption).

The model fit was assessed using the Akaike Information Criterion (AIC = 6216.128), the Bayesian Information Criterion (BIC = 6271.117.768), and the log‐likelihood (LL = −3098.064). Figure 2 reports the plot of the Estimated Marginal Effects of the fixed effects Pasta Consumption and Place (Random effects were plotted across Participants). The significant positive effect of the interaction between pasta consumption and where pasta is consumed on the variation of Happiness suggests that participants who consumed pasta together reported greater increases in happiness compared to those who consumed at home. The random intercept variance underscores the importance of accounting for individual differences in baseline happiness levels. The model fit indices indicate that the model provides a reasonable fit to the data. The no significance of the main fixed effects of pasta consumption or the place where it is consumed indicates that eating pasta significantly increases happiness only when consumed together, rather than at home, alone, or at work, confirming the social aspect of this food in Italian culture.

## 4 Discussion

The findings of this study expand upon the existing knowledge regarding the emotional impact of pasta consumption. Across various methodologies—including self‐reported assessments, implicit association tests, and ecological momentary assessments—participants consistently associated pasta with positive emotions, particularly happiness. The spontaneous associations highlighted family gatherings, warmth, comfort, and Italian traditions as central images linked with pasta. These results align with previous research indicating that foods considered part of the Mediterranean diet, of which pasta is a key component, are often associated with positive emotions and psychological well‐being (Donini et al. 2015; Guasch‐Ferré and Willett 2021). However, to our knowledge, this is the first time the specific association between pasta and happiness, which is a more specific aspect of psychological well‐being, has been explored in depth. The study reveals that pasta consumption does not merely evoke a transient positive emotional response but may have more sustained effects on psychological well‐being. This is suggested by the consistent association between pasta consumption and lower stress levels, higher quality of life, and increased mindfulness. While the correlational nature of this study does not allow for causal conclusions, the data seem to indicate that either pasta consumption contributes to enhanced well‐being or that individuals with higher psychological well‐being are more inclined to appreciate pasta. The strong relationship between pasta appreciation and mindfulness/mindful eating also supports the hypothesis that being mindful enhances the enjoyment and positive emotional impact of pasta. It may reflect the role of present‐moment awareness in enhancing the enjoyment of everyday experiences. Individuals with greater mindfulness disposition may be more attuned to the sensory and emotional qualities of the meal, including taste, texture, and the social atmosphere, deepening the emotional resonance of food‐related experiences and leading to stronger positive affect. Mindfulness may also reduce distractions or judgmental thoughts during meals, allowing for a more immersive and rewarding interaction with food. Future studies could further explore the relationship between pasta consumption, psychological well‐being, and mindfulness.

The emotional impact of pasta consumption primarily manifests in increased levels of happiness, with no or limited changes to the other considered effects and feelings. The ecological momentary assessment indicated that pasta consumption has a significant positive effect on happiness, especially when consumed in social settings. This highlights the importance of the social context in which food is consumed, a factor that might amplify the positive emotions associated with pasta (Bernardi and Visioli 2024). The significant interaction between pasta consumption and the place where it is consumed underscores the cultural and social dimensions of eating practices, particularly in Italy, where communal meals are deeply ingrained in the cultural fabric (Moro and Galletti 2022).

The implicit association tests demonstrated a pronounced positive bias towards pasta, indicating that these positive emotions are not solely conscious reflections but also operate at a subconscious level. This finding is particularly relevant for marketing and health promotion strategies as it suggests that pasta can be positioned as not only a staple of the diet but also as a promoter of emotional well‐being.

The strong emotional response to pasta observed in this study is likely influenced by its deep cultural roots in Italian society, where pasta is often associated with family traditions, social rituals, and national identity (Parasecoli 2025), all of which contribute to its symbolic and emotional weight. These cultural dimensions may help explain the intensity and consistency of the positive emotional responses reported. However, the emotional significance of staple foods can vary widely across cultures (Rozin 2005)—rice in many Asian countries, bread in parts of the Middle East, or corn‐based dishes in Latin America may carry similar symbolic and emotional roles. To explore the universality or specificity of the observed patterns, future research would benefit from cross‐cultural replications, examining whether analogous emotional associations emerge around culturally central foods in different national contexts.

Despite the strengths of this study, some limitations should be considered. Due to the observational nature of the study, causal inferences cannot be drawn. While the results consistently show a positive association between pasta consumption and happiness, it remains unclear whether eating pasta actively contributes to increased happiness or whether individuals who are already happier are more likely to enjoy or choose pasta. Similarly, confounding variables such as general lifestyle factors or the frequency of social engagement may play a role in shaping both eating habits and emotional states. Future studies employing experimental or longitudinal designs would be helpful in clarifying the direction and nature of these effects. The entire sample was drawn from the Italian population, where pasta is not just a common food item but a symbol of cultural identity and tradition. This strong cultural connection may have influenced participants' emotional responses, potentially limiting the extent to which these findings can be generalized to other countries or cultural contexts. Replicating the study in different settings would help determine whether similar associations with pasta emerge elsewhere. Another aspect that was not systematically explored concerns the variety of pasta shapes and especially the wide range of toppings and sauces typically used. It is possible that these variations, which influence both taste and presentation, may also shape the emotional experience of eating pasta. Further research could investigate whether specific types of pasta or culinary preparations are linked to different emotional responses. Additionally, while Study 1 was conducted on a large and nationally representative sample, the second study involved a smaller, self‐selected group of participants, which may have introduced a degree of selection bias. Participants who opted in may differ in relevant psychological or behavioral characteristics from those who did not, potentially affecting the generalizability of the EMA findings. Future research should aim for more inclusive recruitment strategies and larger, stratified samples to enhance external validity. While the social setting was considered, other contextual factors–such as the specific occasion of the meal, perceived food quality, or how the pasta was prepared–were not collected. These situational elements may also influence emotional responses and should be examined in future research. Additionally, the intensity of EMA data collection may have introduced participant fatigue or response bias, potentially affecting data quality. Future studies could consider adjusting prompt frequency to reduce this burden.

## 5 Conclusion

This multi‐method study provides a comprehensive analysis of the emotional associations with pasta consumption, revealing a consistent correlation between pasta and positive emotions, particularly happiness. The data indicate that pasta consumption is frequently linked with images of family, warmth, and comfort, aligning with cultural and advertising narratives in Italy. These findings have important implications for understanding the role of specific foods in emotional well‐being and can inform health promotion strategies and marketing efforts. Pasta's ability to evoke positive emotions and its potential link to improved psychological well‐being suggest that it can be more than just a nutritional choice. This could be leveraged in public health strategies to enhance mental health and well‐being, particularly in promoting social and familial eating contexts. Furthermore, when consumed in a social context, the impact on happiness is particularly pronounced. No other foods, whether carbohydrate‐based meals such as rice, bread, and pizza, or other nutritional options, including meat and junk food, were associated with a similar increase in happiness when consumed in the presence of others. The data suggest that the combination of pasta and social interaction is a significant booster of happiness.

In conclusion, this research contributes to the understanding of how pasta, beyond its nutritional value, is deeply intertwined with emotions, cultural identity, and well‐being. As we continue to explore the complex relationship between food and emotions, these insights pave the way for further research and practical applications in both health promotion and marketing domains. Future studies should aim to establish causal relationships and explore the underlying mechanisms through which pasta and other culturally significant foods influence emotional and psychological well‐being.

## Author Contributions

**F. Grosso:** conceptualization (equal), data curation (equal), investigation (equal), methodology (equal), writing – original draft (equal). **A. Bonanomi:** formal analysis (equal), writing – original draft (supporting). **F. Pagnini:** conceptualization (equal), data curation (equal), funding acquisition (equal), investigation (equal), methodology (equal), supervision (equal), writing – original draft (equal).

## Ethics Statement

This study was conducted in accordance with the ethical standards of the Declaration of Helsinki and was approved by the Ethics Committee of the Department of Psychology at Università Cattolica del Sacro Cuore (ref. 71–23).

## Consent

All participants provided informed consent prior to participation in the study.

## Conflicts of Interest

The authors declare no conflicts of interest.

## Data Availability

The datasets generated and analyzed during the current study are available from the corresponding author on reasonable request. Data sharing will be subject to compliance with ethical and privacy considerations.
